# Prevalence and Impact of Fatigue in Children with Primary Immunodeficiency Disorders: a Quantitative Single-Center Study

**DOI:** 10.1007/s10875-022-01282-w

**Published:** 2022-05-10

**Authors:** Eline Visser, Pieter Fraaij, Annemieke Hoogenboom, Erica Witkamp, Linda van der Knaap, Annemarie van Rossum, Kim Stol, Clementien Vermont

**Affiliations:** 1 Pediatric Infectious Diseases & Immunology, Erasmus MC-Sophia Children’s Hospital, Rotterdam, The Netherlands; 2 School of Health Care Studies, Rotterdam University of Applied Sciences, Rotterdam, The Netherlands

**Keywords:** Fatigue, PedsQL-MFS, primary immunodeficiency disorders, children, quality of life

## Abstract

Although fatigue is a common symptom in adult patients with primary immunodeficiencies (PID), data in pediatric patients are limited. The goal of this study is to estimate the prevalence and impact of fatigue in children with PID as reported by patients, parents, and health-care providers. A retrospective single-center observational study was performed. Prevalence of fatigue was measured by reviewing medical charts of 54 children in our department who are on immunoglobulin replacement therapy. Both prevalence and impact were also measured by the PedsQL-Multidimensional Fatigue Scale (MFS) in 27 patients and 32 of their parents. This is an age-appropriate questionnaire for self-report of fatigue symptoms in patients aged 5–18 years and for parent proxy reports for patients aged 2–18 years. General, cognitive, and sleep-rest fatigue was measured, and a total fatigue score was calculated. Means, standard deviation and *Z* scores were calculated using age-specific reference values. Intraclass correlation coefficients (ICC) were calculated for comparison of scores provided by parents vs children’s self-reported scores. Both chart review data and PedsQL-MFS showed fatigue rates of 65%. Pediatric PID patients of all ages had significantly lower scores on all subscales and total score of the PedsQL-MFS compared to healthy children, indicating greater perceived symptoms of fatigue. General fatigue was the most affected subscale in PID patients, suggesting that fatigue in these patients is mainly physical. Seventy-four percent of PID patients had a *Z* score lower than − 1 on the general fatigue subscale indicating severe fatigue. Child-parent concordance varied between 0.24 and 0.93. Our results show the feasibility of the PedsQL-MFS survey to evaluate the prevalence and severity of fatigue in children with PID and underscore the importance of this issue in our patient care.

## Introduction

Primary immunodeficiency disorders (PID) represent a group of over 400 rare disorders affecting the immune system [1]. The exact prevalence of PID is not known, but in 2013, there were an estimated 6 million people living with PID worldwide [2]. Children with PID often present with recurrent or chronic bacterial infections of the upper or lower respiratory tract (e.g., otitis media, sinusitis, pneumonia), diarrhea, and “failure to thrive” [3]. Over the years, there have been major improvements in the clinical care of PID patients [4], especially due to improved awareness, earlier diagnosis, and appropriate immunoglobulin (IgG) therapies that led to increased life expectancy [5–7]. Despite these improvements, patients suffering from PID still report a poorer health-related quality of life compared to healthy controls [8, 9]. They even report a worse quality of life compared to patients suffering from other chronic conditions such as diabetes and juvenile idiopathic arthritis [10]. Seeborg et al. (11) showed that over a third of the PID patients perceived their health status as fair or poor to very poor with fatigue as an important factor [11]. Severe fatigue contributes to impaired functional status, reduced school participation, depression, and poor health-related quality of life [12–14]. Fatigue does not only affect the patient, but also has great impact on their family [13, 15, 16].

A large study on fatigue in patients with PID was conducted by Hajjar et al. [17]. A total of 2537 PID patients registered in an American database were analyzed in order to identify associations and potential drivers for fatigue. They found several indicators associated with higher rates of reported fatigue: a diagnosis of common variable immune deficiency (CVID), female sex, high body mass index, depression, bronchiectasis, and autoimmunity. A small part of the study population consisted of children: 19 children were under the age of 8 years and 156 children between the age of 9 and 18 years. Fatigue in this study was scored as present when it was checked (yes/no) as a constitutional symptom in the patient registry form of the database. No differentiation in fatigue symptoms or parents’ impressions were taken in consideration. Other studies showed that in patients with PID, fatigue is more frequently reported by patients who receive intravenous immunoglobulin treatment in comparison to patients who receive subcutaneous infusions [18, 19]. These feelings of exhaustion are especially present immediately prior to the patient's next infusion [20]. This is called the “wear-off effect” and is thought to be caused by low trough levels of IgG [21, 22]. Nowadays, the vast majority of our pediatric PID patients receive subcutaneous immunoglobulin infusion, which provide steady IgG levels and might result in less fatigue.

The purpose of our study is to determine the prevalence of fatigue specifically in pediatric PID patients treated with immunoglobulin replacement. Chart data were reviewed for patient characteristics, and a validated pediatric fatigue questionnaire in patients as well as their parents was used to compare severity of fatigue to healthy peers and children with other chronic diseases.

## Methods

### Inclusion and Exclusion Criteria

Children with a diagnosis of hypogammaglobulinemia, CVID, or X-linked agammaglobulinemia (XLA) currently on immunoglobulin replacement therapy in our hospital were included in the study. Patients and parents who did not have sufficient Dutch language skills to read the questionnaire were excluded (Table 1).

**Table 1:** Characteristics of the eligible population of children with PID using immunoglobulin treatment

	Chart review	Survey	
		Eligible	Responders
	*N* = 56	*N* = 50	*N* = 32
Male (%)	36 (64%)	33 (66%)	20 (63%)
Female (%)	20 (36%)	17 (34%)	12 (37%)
Mean age in years (range)	11.5 (1–18)	11.2 (2–17)	10.7 (4–17)
IVIG	13 (23%)	11 (32%)	4 (13%)
f-SCIG	17 (31%)	16 (32%)	10 (31%)
SCIG	26 (46%)	23 (46%)	18 (56%)
CVID	15 (27%)	11 (22%)	4 (13%)
Hypogammaglobulinemia	26 (47%)	25 (50%)	19 (59%)
SPAD	5 (9%)	5 (10%)	4 (13%)
X-linked agammaglobulinemia	4 (7%)	4 (8%)	2 (6%)
Other antibody deficiency	6 (11%)	5 (10%)	3 (9%)
BMI > + 2 SD	11 (20%)	9 (18%)	6 (19%)
Bronchiectasis on CT	17 (30%)	15 (30%)	7 (22%)
Psychological support	20 (36%)	18 (36%)	14 (44%)
Autoimmune symptoms	15 (27%)	15 (30%)	8 (25%)

The data obtained from the chart review were compared to the respondents of the postal survey

*CVID* common variable immunodeficiency, *f-SCIG* facilitated-SCIG, *IVIG* intravenous immunoglobulin, *SPAD* specific polysaccharide antibody deficiency, *BMI* body mass index

### Procedures

The first phase of our study was a chart review to repeat the research of Hajjar et al. in our pediatric population. All medical and nursing consultations between December 1, 2015, and December 1, 2017, were reviewed. Physicians and nurses have not been instructed to ask actively for fatigue in that period. Binary scores (yes/no) of fatigue were assigned when chart reviews revealed complaints of fatigue, and additional information such as sex, age, and diagnosis was collected on the Case Record Form. Furthermore, hemoglobulin levels and thyroid-stimulating hormone (TSH) when available in the review period were scored and determined normal or abnormal according to age-matched reference values.

The second phase consisted of a postal survey using the PedsQL™ MFS in children aged 2–18 years in order to quantify the presence and extent of fatigue among children with PID who use immunoglobulin treatment on the index date April 1, 2018. A child self-report as well as one parent’s proxy report was conducted.

### Measures

The PedsQL™ MFS is a generic questionnaire composed of 18 items comprising three subscales: General Fatigue (GF), Sleep-Rest Fatigue (SRF), and Cognitive Fatigue (CF). Patients rated their problems over the past 4 weeks, using a 5-point Likert scale from 0 (never) to 4 (almost always). For the child report for young children (age 5 to 7 years), there is a 3-point scale: 0 (not at all), 2 (sometimes), and 4 (a lot). The Dutch translation of the PedsQL™ MFS was previously validated in healthy children aged 2 to 18 years old [23]. The scores obtained in these healthy children were used as reference values. In our population of pediatric PID patients using immunoglobulin, each item was reverse scored and linearly transformed to a 0–100 scale as follows: 0 = 100, 1 = 75, 2 = 50, 3 = 25, 4 = 0. The scale score is the sum of the items over the number of items answered. Lower scores indicate greater perceived symptoms of fatigue. The total fatigue score (TFS) is the mean of the 3 subscale scores (GF, SRF, and CF) and is calculated for patients and parents separately. Patients as well as their parents were asked to fill in a questionnaire. Scores were converted into standardized *Z* scores for comparison to reference values using means and standard deviation for each age group provided by Gordijn et al. [23]. A *Z* score on any scale or on the TFS lower than − 1 was considered abnormal (more fatigued compared to healthy peers).

### Statistical Analysis

Analyses were performed using SPSS 25.0 (IBM, Chicago, IL, USA). The Kolmogorov–Smirnov test was used to test whether data were normally distributed. Results of the binary score on fatigue symptoms based on the chart review were compared with a *Z* score equal to or less than − 1 on the General Fatigue scale and the TFS of the PedsQL™ MFS as provided by parents (proxy report) and patients (self-report) using the McNemar test. For comparison between self and proxy report on the PedsQL™ MFS, intraclass correlation coefficients (ICC) were calculated. One-sample *t*-tests were used when comparing *Z* scores of patients with the reference group (test value = 0 or − 1). Independent sample *T* tests were used for group comparisons. A *p* value of < 0.05 was considered as being significant.

### Ethical Considerations

The study protocol was approved by the Institutional Review Board of Erasmus MC, the Netherlands (MEC-2018–1161). All patients and/or parents provided written informed consent.

## Results

### Patient Inclusion and Characteristics

**Fig. 1:**
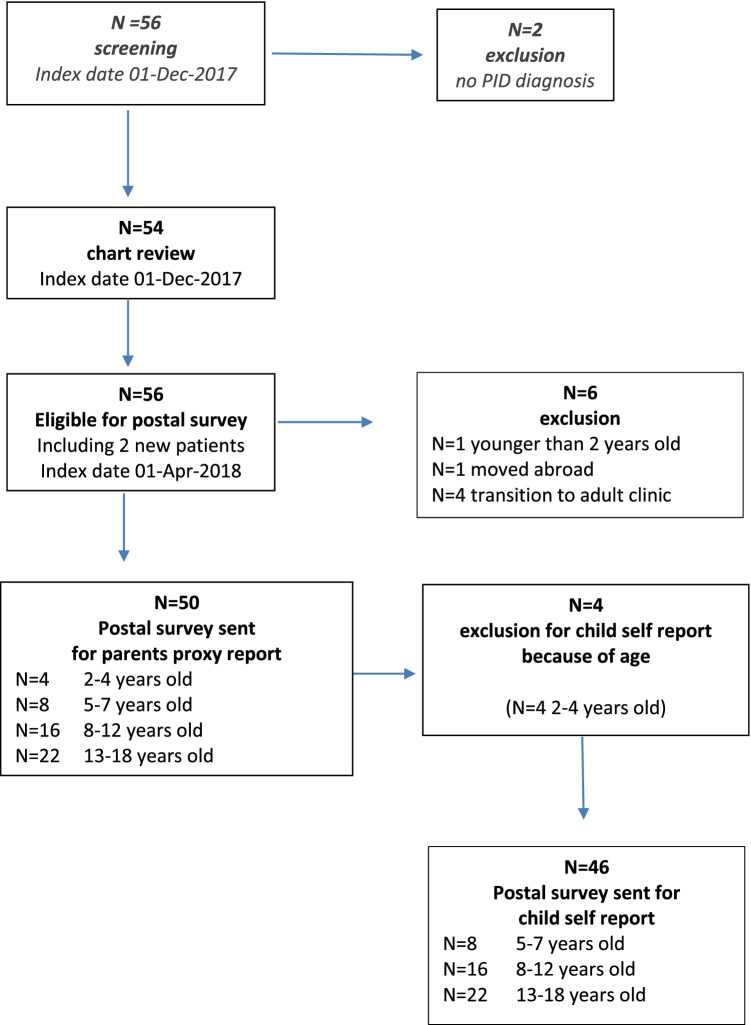
Flowchart of screening and inclusion

After screening all patients on IgG replacement therapy (*n* = 56), 2 patients were excluded from our study because of using IgG therapy for a neurological diagnosis. All 54 eligible patients were included in the chart review. For the postal survey, 56 patients on immunoglobulin replacement therapy were eligible on the index date (see Fig. 1). Forty-six child questionnaires and 50 parent questionnaires were distributed by mail, of which 27 and 32 were returned, respectively (response rates 59 and 64%, Fig. 1). No significant differences in patient characteristics were found between responders and non-responders. In total, 1062 items (27 children and 32 parents × 18 MFS™-items) were scored; 4 children missed a score on one item, and one parent failed to fill out four items (missing item response rate: 0.8%). Sociodemographic characteristics are presented in Table 1. The mean age of the respondents was 10.7 years. Ninety-one percent of the responders had normal values for hemoglobulin levels. TSH was abnormal in 2 patients; however, in 53% of the patients TSH was not measured on consultation (data not shown). Hypogammaglobulinemia and CVID were the most common conditions in our population, and 77% of the patients received IgG replacement via the subcutaneous route. The duration of immunoglobulin treatment was ≤ 2 years in 39% of the patients, whereas 43% of the patients have been treated with Ig therapy ≥ 5 years.

The chart review revealed that 64% of all children with PID on IgG replacement therapy reported symptoms of fatigue in one of the consultations during the study period of 2 years. These binary scores (fatigue yes/no) were compared to the self-reported scores of patients on the subscale of General Fatigue (GF) and to the Total Fatigue Score (TFS). The chart review binary score corresponded well with a *Z* score <  − 1 on the GF scale as scored by patients as well as their parents (McNemar test non-significant *p* values) but not with a self-reported *Z* score <  − 1 on the Total Fatigue score (McNemar *p* = 0.021).

### PedsQL™ Multidimensional Fatigue Scale

All scores of the PedsQL™ Multidimensional Fatigue Scale (MFS) in the various age groups are summarized in Table 2 and compared with reference values. PID patients had overall lower scores on all scales when compared to the healthy reference group, especially on the general fatigue scale. In general, the scores of children and parents were in concordance, although children suffering from PID scored themselves slightly higher (less fatigue) than their parents (Table 2). *Z* scores were calculated to correct for standard deviation and age as is shown in Table 3. General fatigue and TFS *Z* scores were significantly lower in every age group and in the total group. General fatigue *Z* scores of the total group in both self- and proxy reports were lowest: − 1.78 and − 2.45, respectively.

**Table 2:** PedsQL™ Multidimensional Fatigue Scale (MFS) scores on the Total Fatigue Scale (TFS) and the separate subscales: General Fatigue (GF), Sleep-Rest Fatigue (SRF), and Cognitive Fatigue (CF)

	2–4 years		5–7 years		8–12 years		13–18 years	
Self-report	Study Mean (SD)	REF	Study *N* = 6 Mean (SD)	REF *N* = 68 Mean (SD)	Study *N* = 11 Mean (SD)	REF *N* = 143 Mean (SD)	Study *N* = 10 Mean (SD)	REF *N* = 155 Mean (SD)
Total fatigue score	NA	NA	56.48 (10.92)	76.59 (14.19)	58.75 (20.57)	78.70 (12.50)	61.53 (18.89)	75.24 (11.95)
General fatigue	NA	NA	58.33 (18.26)	83.46 (15.89)	56.06 (22.00)	82.66 (12.92)	53.75 (24.33)	76.72 (14.31)
Sleep-rest fatigue	NA	NA	59.72 (16.17)	74.00 (18.68)	62.08 (18.05)	77.55 (15.04)	57.50 (21.94)	71.88 (14.17)
Cognitive fatigue	NA	NA	51.39 (16.17)	72.24 (21.69)	65.53 (30.28)	75.76 (19.13)	73.33 (22.84)	77.15 (15.30)
Proxy — report	*N* = 2 Mean (SD)	REF *N* = 104 Mean (SD)	*N* = 6 Mean (SD)	REF *N* = 83 Mean (SD)	*N* = 13 Mean (SD)	REF *N* = 149 Mean (SD)	*N* = 11 Mean (SD)	REF *N* = 161 Mean (SD)
Total fatigue score	68.06 (1.96)	82.87 (10.76)	48.84 (11.63)	83.01 (11.35)	54.75 (18.55)	81.25 (12.73)	52.92 (22.84)	79.17 (13.99)
General fatigue	70.83 (0.00)	82.80 (11.84)	48.61 (13.09)	84.46 (12.35)	43.27 (12.56)	82.27 (13.95)	46.97 (25.62)	77.71 (15.93)
Sleep-rest fatigue	54.17 (29.46)	82.92 (13.17)	55.56 (16.60)	87.77 (12.19)	57.69 (18.23)	85.49 (13.21)	51.67 (21.35)	80.87 (15.06)
Cognitive fatigue	79.17 (23.57)	82.77 (15.09)	42.36 (24.07)	76.71 (17.18)	62.15 (32.25)	75.98 (19.61)	65.53 (27.83)	78.93 (17.99)

*REF* reference group, healthy peers from the study of Gordijn et al. [23]

**Table 3:** *Z* scores of all participants on every PedsQL MFS as reported by patients themselves

PedsQL Multidimensional Fatigue Scale	5–7 years *N* = 6	8–12 years *N* = 11	13–18 years *N* = 10	Total group *N* = 27
Self-report	Mean *Z* score (SD)	Mean *Z* score (SD)	Mean *Z* score (SD)	Mean *Z* score (SD)
General fatigue	− 1.58 (1.15)*	− 2.06 (1.70)*	− 1.61 (1.70)*	− 1.78 (1.56) **
Cognitive fatigue	− 0.96 (0.75)*	− 0.53 (1.58)	− 0.25 (1.49)	− 0.52 (1.38)*
Sleep-rest fatigue	− 0.76 (0.87)	− 1.03 (1.20)*	− 1.01 (1.55)	− 0.96 (1.24)*
Total fatigue	− 1.42 (0.77)*	− 1.60 (1.65)*	− 1.15 (1.58)*	− 1.38 (1.43)*
Proxy-report	*N* = 6	*N* = 12	*N* = 10	*N* = 28
General fatigue	− 2.90 (− 2.06)**	− 2.79 (0.94)**	− 2.05 (1.64)**	− 2.45 (1.28)**
Cognitive fatigue	− 2.00 (1.40)*	− 0.71 (1.64)	− 0.94 (1.49)	− 1.01 (1.56)*
Sleep-rest fatigue	− 2.64 (1.36)**	− 2.03(1.41)**	− 1.94(1.42)**	− 2.13 (1.39)**
Total fatigue	− 3.01(1.03)**	− 2.08 (1.46)**	− 1.88 (1.63)*	− 2.15 (1.43)**

A negative *z* score indicates more fatigue symptoms than the reference group

^*^ indicates a significant difference as tested by one-sample t-test (test-value = 0); ** indicates a significant difference as tested by one-sample *t*-test (test value =  − 1)

There were no differences in *Z* scores between male and female patients or between the different age groups (data not shown). Twenty out of twenty-seven (74%) patients had a *Z* score <  − 1 on the GF scale, indicating significantly more fatigue than their healthy peers. Thirteen out of twenty-seven (48%) had a *Z* score of <  − 1 on the TFS.

In young children, self-reported vs proxy scores were profoundly different: intraclass correlation coefficient (ICC) of 0.24 in the 5–7-year group, whereas scores of 8–12 years and 13–18-year-olds and their parents were fairly similar (both groups ICC 0.93; Table 4).

**Table 4:** Child parent concordance measured by Intraclass Correlation Coefficient (ICC) for PedsQL™ Multidimensional Fatigue Scale (MFS) on the total MFS™ score and on the separate subscales: General Fatigue (GF), Sleep-Rest Fatigue (SRF) and Cognitive Fatigue (CF)

	2–4 years		5–7 years		8–12 years		13–18 years		Total group	
	ICC *N* = NA	ICC REF *N* = NA	ICC *N* = 6	ICC REF *N* = 68	ICC *N* = 11	ICC REF *N* = 140	ICC *N* = 10	ICC REF *N* = 153	ICC *N* = 32	REF ICC *N* = 361
TFS	NA	NA	0.24	0.25	0.93	0.68	0.93	0.52	0.85	0.53
GF	NA	NA	0.31	0.22	0.43	0.48	0.90	0.50	0.61	0.44
SRF	NA	NA	0.51	0.10	0.90	0.46	0.91	0.41	0.83	0.36
CF	NA	NA	0.74	0.22	0.87	0.66	0.91	0.45	0.87	0.49

*TFS* total fatigue score, *ICC* intraclass correlation coefficient (ICC), *ICC REF* intraclass correlation coefficient (ICC) for the reference group, healthy peers form the study of Gordijn et al. [23]

## Discussion

Our study measures the prevalence and severity of fatigue in children with PID as reported by themselves as well as by their parents/caregivers during medical consultations and PedsQL-MFS questionnaires. Children with PID have lower scores on nearly all subscales compared to the reference group, meaning they are more fatigued than their healthy peers. This is especially obvious concerning General Fatigue (GF): a mean *Z* score of − 1.78. The chart review–based prevalence of fatigue in our pediatric PID population on IgG replacement therapy was 64%. This is a significantly higher prevalence than reported in the American database study of Hajjar et al. who showed a prevalence of 26% of fatigue in US patients with primary antibody deficiencies [17] and comparable with adult CVID patient reports (76.9%) [21].

The chart review–based prevalence of fatigue corresponded well with *Z* scores less than − 1 on the general fatigue score of the PedsQL*™* as scored by self-assessment as well as by proxy report by parents. Remarkably, our patients generally score worse on fatigue than pediatric patients with other severe chronic diseases such as multiple sclerosis and childhood cancer, emphasizing the high burden of fatigue symptoms in our patients [24, 25]. A possible explanation for this could be the chronic inflammation which is associated with CVID, or other PID-associated comorbidities such as bronchiectasis, pulmonary granuloma, or autoimmune diseases.

Literature on CVID patients also report fatigue as a result of declining trough levels of IgG in patients who are on intravenous IgG replacement therapy [21]. However, the vast majority of patients in our study receive SCIG, which provides a fairly stable IgG level during treatment. Fatigue symptoms in our patients therefore cannot be explained by low trough levels or other therapeutic factors.

According to other studies, a diagnosis of CVID, higher age, female gender, having a high BMI, depression, bronchiectasis, autoimmunity, and receiving IVIG treatment are factors associated with fatigue [17–19]. These risk factors and their prevalence in our study population were evenly distributed among the chart review and the eligible population of the survey as well as between responders and non-responders to the survey. However, the sample size of our study was still too small to assess whether these are significant factors contributing to fatigue.

Fatigue is a multidimensional concept, in which not only medical but also psychological and social factors play a role. We found no obvious medical explanation such as anemia or hypothyroidism for fatigue, but have no information on psychiatric comorbidity. Depression can cause significant fatigue symptoms and can be difficult to diagnose in young children. Use of other age-specific pediatric measurement tools simultaneously may be helpful to evaluate psychological causes of fatigue in PID patients. Emotional distress of having a chronic disease and socio-economic factors may also contribute to fatigue symptoms. Children with PID have the lowest scores on the GF subscale and the highest scores on CF, indicating their type of fatigue is mainly physical. Studies performed in children with other chronic conditions such as sickle cell disease and multiple sclerosis do not report such a difference [24, 26]. A potential clinical implication of this finding might be the development of exercise treatment programs for children with PID, to improve their condition in general and a feeling of well-being despite having a chronic disease. This is not yet standard of care for children with PID and warrants further research. Using PedsQL MFS survey routinely during follow-up at the outpatient clinic would help to see if any therapeutic interventions, medical as well as psychological, influence fatigue symptoms in PID patients.

Our observations on the child-parent concordance differ from the findings in literature. In health-related quality-of-life measurements, there usually is a low child-parent concordance with lower scores reported by the children, whereas our results showed higher scores on all subscales in children compared to the proxy report [23, 27]. In our study, the concordance between teenagers and parents was relatively high (0.90–0.93). In the 5–7-year-age group, it was much lower (0.24–0.74). These results indicate that patients in this age group filled out the questionnaires independent from their parents. It may also indicate that the questionnaire is complex for younger children. Furthermore, it may represent parents’ concerns about their child wellbeing, scoring them far more fatigue than they do themselves. 

The present study has several strengths. To our knowledge it is the first study using PedsQL MFS surveys in children with PID; it represents a broad age range of participants and was valid and reliable. The external validity of the postal survey study was warranted by a reasonably high response rate and by using reference values of a large cohort of healthy Dutch peers. A small number of patients scored extremely high or extremely low. The minimum self-reported Total Fatigue Score (TFS) was 29, whereas the maximum was 94. These so-called floor and ceiling effects make response bias unlikely and indicate a strong reliability.

Limitations include the small sample size. The prevalence of known risk factors of fatigue in PID patients was too low to analyze. Fatigue as a symptom was not routinely asked for on consultations, which leads to a reporting bias in the chart review scores. We measured fatigue scores at only one timepoint. To rule out possible seasonal influences, a future study would need to have at least one timepoint in spring and one in fall.

In conclusion, the results of our study show the presence of severe fatigue in a majority of pediatric PID patients on immunoglobulin replacement therapy. The PedsQL MFS survey is a feasible tool to screen and identify severely fatigued PID patients and their caretakers’ impression. Studies on effective interventions on fatigue are warranted and should be investigated in this patient group to further improve quality of life and well-being.

## Data Availability

The dataset described in this study is available from the corresponding author upon reasonable request.

## References

[1] Bousfiha A, Jeddane L, Picard C, Al-Herz W, Ailal F, Chatila T (2020). Human inborn errors of immunity: 2019 Update of the IUIS Phenotypical Classification. J Clin Immunol.

[2] Bousfiha AA, Jeddane L, Ailal F, Benhsaien I, Mahlaoui N, Casanova JL (2013). Primary immunodeficiency diseases worldwide: more common than generally thought. J Clin Immunol.

[3] de Vries E, Driessen G (2011). Educational paper: primary immunodeficiencies in children: a diagnostic challenge. Eur J Pediatr.

[4] Chapel H, Prevot J, Gaspar HB, Espanol T, Bonilla FA, Solis L (2014). Primary immune deficiencies - principles of care. Front Immunol.

[5] Busse PJ, Razvi S, Cunningham-Rundles C (2002). Efficacy of intravenous immunoglobulin in the prevention of pneumonia in patients with common variable immunodeficiency. J Allergy Clin Immunol.

[6] Orange JS, Hossny EM, Weiler CR, Ballow M, Berger M, Bonilla FA (2006). Use of intravenous immunoglobulin in human disease: a review of evidence by members of the Primary Immunodeficiency Committee of the American Academy of Allergy, Asthma and Immunology. J Allergy Clin Immunol.

[7] Resnick ES, Moshier EL, Godbold JH, Cunningham-Rundles C (2012). Morbidity and mortality in common variable immune deficiency over 4 decades. Blood.

[8] Jiang F, Torgerson TR, Ayars AG (2015). Health-related quality of life in patients with primary immunodeficiency disease. Allergy Asthma Clin Immunol.

[9] Sultan S, Rondeau E, Levasseur MC, Dicaire R, Decaluwe H, Haddad E (2017). Quality of life, treatment beliefs, and treatment satisfaction in children treated for primary immunodeficiency with SCIg. J Clin Immunol.

[10] Peshko D, Kulbachinskaya E, Korsunskiy I, Kondrikova E, Pulvirenti F, Quinti I (2019). Health-related quality of life in children and adults with primary immunodeficiencies: a systematic review and meta-analysis. J Allergy Clin Immunol Pract..

[11] Seeborg FO, Seay R, Boyle M, Boyle J, Scalchunes C, Orange JS (2015). Perceived health in patients with primary immune deficiency. J Clin Immunol.

[12] Crichton A, Knight S, Oakley E, Babl FE, Anderson V (2015). Fatigue in child chronic health conditions: a systematic review of assessment instruments. Pediatrics.

[13] Huang IC, Anderson M, Gandhi P, Tuli S, Krull K, Lai JS (2013). The relationships between fatigue, quality of life, and family impact among children with special health care needs. J Pediatr Psychol.

[14] Viner RM, Clark C, Taylor SJ, Bhui K, Klineberg E, Head J (2008). Longitudinal risk factors for persistent fatigue in adolescents. Arch Pediatr Adolesc Med.

[15] Harris K, Band RJ, Cooper H, Macintyre VG, Mejia A, Wearden AJ (2016). Distress in significant others of patients with chronic fatigue syndrome: a systematic review of the literature. Br J Health Psychol.

[16] van't Leven M, Zielhuis GA, van der Meer JW, Verbeek AL, Bleijenberg G. Fatigue and chronic fatigue syndrome-like complaints in the general population. Eur J Public Health. 2010;20(3):251–7. 10.1093/eurpub/ckp113 19689970

[17] Hajjar J, Guffey D, Minard CG, Orange JS (2017). Increased incidence of fatigue in patients with primary immunodeficiency disorders: prevalence and associations within the US Immunodeficiency Network Registry. J Clin Immunol.

[18] Misbah SA (2014). Effective dosing strategies for therapeutic immunoglobulin: managing wear-off effects in antibody replacement to immunomodulation. Clin Exp Immunol.

[19] Gustafson R, Gardulf A, Hansen S, Leibl H, Engl W, Linden M (2008). Rapid subcutaneous immunoglobulin administration every second week results in high and stable serum immunoglobulin G levels in patients with primary antibody deficiencies. Clin Exp Immunol.

[20] Rider NL, Kutac C, Hajjar J, Scalchunes C, Seeborg FO, Boyle M (2017). Health-related quality of life in adult patients with common variable immunodeficiency disorders and impact of treatment. J Clin Immunol.

[21] Hajjar J, Kutac C, Rider NL, Seeborg FO, Scalchunes C, Orange J (2018). Fatigue and the wear-off effect in adult patients with common variable immunodeficiency. Clin Exp Immunol.

[22] Rojavin MA, Hubsch A, Lawo JP (2016). Quantitative evidence of wear-off effect at the end of the intravenous IgG (IVIG) dosing cycle in primary immunodeficiency. J Clin Immunol.

[23] Gordijn M, Cremers EM, Kaspers GJ, Gemke RJ (2011). Fatigue in children: reliability and validity of the Dutch PedsQL Multidimensional Fatigue Scale. Qual Life Res.

[24] Toussaint-Duyster LC, Wong YYM, Van der Cammen-van Zijp MH, Van Pelt-Gravesteijn D, Catsman-Berrevoets CE, Hintzen RQ (2018). Fatigue and physical functioning in children with multiple sclerosis and acute disseminated encephalomyelitis. Mult Scler.

[25] Varni JW, Burwinkle TM, Katz ER, Meeske K, Dickinson P (2002). The PedsQL in pediatric cancer: reliability and validity of the Pediatric Quality of Life Inventory Generic Core Scales, Multidimensional Fatigue Scale, and Cancer Module. Cancer.

[26] Panepinto JA, Torres S, Bendo CB, McCavit TL, Dinu B, Sherman-Bien S (2014). PedsQL Multidimensional Fatigue Scale in sickle cell disease: feasibility, reliability, and validity. Pediatr Blood Cancer.

[27] Eiser C, Morse R (2001). Can parents rate their child’s health-related quality of life? Results of a systematic review. Qual Life Res.

